# Highly Superior Autobiographical Memory (HSAM): A Systematic Review

**DOI:** 10.1007/s11065-024-09632-8

**Published:** 2024-02-23

**Authors:** Jessica Talbot, Gianmarco Convertino, Matteo De Marco, Annalena Venneri, Giuliana Mazzoni

**Affiliations:** 1 Faculty of Medicine and Psychology, University La Sapienza, Via Degli Apuli, 00185 Rome, Italy; 2 Department of Life Sciences, Brunel University London, Uxbridge, UK; 3 Department of Medicine and Surgery, University of Parma, Parma, Italy; 4 Department of Psychology, University of Hull, Hull, UK

**Keywords:** Highly Superior Autobiographical Memory, HSAM, Autobiographical memory, Exceptional memory, Systematic review, PRISMA

## Abstract

Individuals possessing a Highly Superior Autobiographical Memory (HSAM) demonstrate an exceptional ability to recall their own past, excelling most when dates from their lifetime are used as retrieval cues. Fully understanding how neurocognitive mechanisms support exceptional memory could lead to benefits in areas of healthcare in which memory plays a central role and in legal fields reliant on witnesses’ memories. Predominantly due to the rareness of the phenomenon, existing HSAM literature is highly heterogenous in its methodologies used. Therefore, following Preferred Reporting Items for Systematic Reviews and Meta-Analyses (PRISMA) guidelines, we performed the first systematic review on this topic, to collate the existing behavioural, neuroanatomical, and functional HSAM data. Results from the 20 experimental selected studies revealed that HSAM is categorised by rapidly retrieved, detailed and accurate autobiographical memories, and appears to avoid the normal aging process. Functional neuroimaging studies showed HSAM retrieval seems characterised by an intense overactivation of the usual autobiographical memory network, including posterior visual areas (e.g., the precuneus). Structural neuroanatomical differences do not appear to characterise HSAM, but altered hippocampal resting-state connectivity was commonly observed. We discuss theories of HSAM in relation to autobiographical encoding, consolidation, and retrieval, and suggest future directions for this research.

**Supplementary Information:**

The online version contains supplementary material available at 10.1007/s11065-024-09632-8.

## Introduction

Highly Superior Autobiographical Memory (HSAM) is a rare form of exceptional memory characterised by an enhanced ability to remember autobiographical content (LePort et al., 2012; Patihis et al., 2013). Internal or external cues, including dates from one’s life span (e.g., 1st January 1999) can elicit HSAM individuals to access specific memories from nearly every day of their past (Gibson et al., 2022; Parker et al., 2006). The skill also involves a remarkable ability to locate memories temporally; participants can accurately and confidently report exact time-related details (e.g., day of the week) of events of their own life and public events of which they have a personal recall (Ford et al., 2022; Parker et al., 2006). HSAM is exclusive to autobiographical memory (ABM), and retrieval is accurate (Ally et al., 2013) and extensively detailed (LePort et al., 2016).

Parker et al. (2006) reported the first case of a woman, given the pseudonym “AJ”, with near perfect ABM, though as far back at the nineteenth century an individual was described possessing similar memory traits (Henkle, 1871). The seminal 2006 study coined the term “hyperthymesia”, referring to the Greek word for remembering (thymesis). At 34 years old, AJ wrote to researchers in California describing a “non-stop, uncontrollable and totally exhausting” ability to remember. When researchers invited her to the laboratory she excelled at numerous standardised and ad hoc ABM tasks, effortlessly providing clear and verifiable memories in response to dates. Since AJ, almost one hundred more individuals have been identified possessing a hyper memory, and the term has been redefined to its more commonly used label of HSAM which reflects its specificity in memory type (Patihis, 2015).

Unlike other forms of exceptional memory, such as Memory Athletes (Dresler et al., 2017), those with HSAM do not utilise deliberate mnemonic techniques (e.g., method of loci) to support encoding or retrieval of information (LePort et al., 2012; Santangelo et al., 2021). Instead, memories are described as entering one’s mind in an automatic way (Mazzoni et al., 2019), and are retained regardless of perceived importance or emotional saliency (Santangelo et al., 2018). The enhanced ability typically manifests during late childhood (De Marco et al., 2021), though for some individuals their ability to remember in excess reportedly begins at 5 years old (Patihis, 2015). The seemingly spontaneous and heightened nature of HSAM makes it a particularly fascinating cognitive phenomenon.

For decades, scientists have investigated the complexity of human memory, but the exact mechanisms of different subtypes are not yet fully understood (Santangelo et al., 2022). HSAM provides a unique angle to explore ABM with potential applications benefitting health and legal contexts. Memory typically involves vast amounts of unintentional forgetting (Maxcey et al., 2019) and is susceptible to age-related cognitive decline (Wright et al., 2021), neurodegeneration, or clinical abnormalities, including mild cognitive impairment or Alzheimer’s Disease (Venneri et al., 2011). Extreme memory impairments negatively impact longevity (Rhodius-Meester et al., 2018), quality of life (Burks et al., 2021), and increase financial burden on healthcare services (Dauphinot et al., 2022). Similarly, misremembering, false memories, or forgetfulness can implicate settings reliant on personal testimonies throughout the justice process (Conway, 2012). Ultimately, by ascertaining neural processes responsible for near-perfect memory, strategies could be implemented to improve normal memory, or to overcome issues of flawed memory.

### Objective

Due to the small population, HSAM research remains relatively scarce. However, researchers generally share the same overarching goals: to understand what people with HSAM are capable of and how they are capable of superior memory. The existing studies have utilised a broad range of paradigms to measure memory and cognitive functioning in HSAM. To our knowledge, no review has systematically organised the available data. We attempt to address this gap and achieve the following objectives. Firstly, we seek to summarise the defining characteristics of HSAM, by collating knowledge from neuropsychological, neuroanatomical, and functional neuroimaging assessments. Secondly, we theorise what this data tells us about the mechanisms supporting HSAM and discuss future directions for this area.

## Methodology

This systematic review adhered to the Preferred Reporting Items for Systematic Reviews and Meta-Analyses (PRISMA) guidelines (Moher, 2009). The protocol was preregistered, details of which can be viewed at https://www.crd.york.ac.uk/PROSPERO/ (ID: CRD42022312854).

### Eligibility Criteria

Full-text articles that reported group or single-cases possessing HSAM or hyperthymesia were selected for this systematic review. No restrictions were made regarding race, age, or sex. Samples that were inadequately screened for HSAM, or who possessed ‘normal’ or dysfunctional memory (e.g., severely deficient autobiographical memory (SDAM)), were excluded. Book chapters, non-English language, non-peer-reviewed, or non-experimental articles were excluded.

### Information Sources and Search Strategy

The first author ran the first of three systematic online literature searches, initially spanning the 1st of January 2006–17th January 2022 on international databases: Web of Science, Scopus, PubMed, Ovid Medline, EBSCO host, and ProQuest. Multiple databases were chosen as this allows literature searches to be thorough (Bramer et al., 2017) and is recommended by gold-standard systematic review guidelines (Lefebvre et al., 2022). Start date was chosen because the HSAM phenomenon was first described that year (Parker et al., 2006). To identify HSAM studies, a search string was devised using advanced search techniques, such as Boolean operators (e.g., OR) and truncations (e.g., hyperthym*, autobiograp*), in the title and abstract fields. The search strings included the following words: (“superior” OR “exceptional” OR “extraordinary” OR “savant” OR “hyperthym*”) AND (“autobiograph*” OR “personal” OR “hyperthym*”) AND (“memor*” OR “retriev*” OR “recall*” OR “recogn*” OR “encod*” OR “rememb*” OR “mnem*” OR “mnes*” OR “recollect*) (Supplementary Materials). Similar strings were used across databases but adapted for each search engine’s specifications. Search strings were rerun approximately six months (1st of January 2006–23rd August 2022) and one year later (1st of January 2006–1st February 2023), to update the pool of eligible manuscripts with the most recent publications. Reference lists from included articles were screened for additional suitable articles. A total of 11,516 results were identified using the strategies described.

### Selection Process

The entire selection process was completed independently by the first and second authors using the predetermined eligibility criteria. Duplicates were removed manually, then titles and abstracts screened. Suitable full-text articles were downloaded and assessed for inclusion. If inclusion agreement could not be reached, the senior researcher, and fifth author, was consulted to make the final decision, using the same eligibility criteria previously described.

### Data Collection

An extraction template was designed on Microsoft Excel, and a pilot of three studies was performed to test appropriateness. Adjustments were made to improve the template design; then key information was extracted by the first and second authors independently. All responses were compared for accuracy verification. Data extracted included article information (location, study aims, and methods) and HSAM population details (sample size, sex, age, handedness, case abbreviation, clinical information, and HSAM screen). In addition, the following results were extracted: behaviour results (task name, purpose of task, and main findings), main structural and functional neuroimaging results (implicated brain areas, neuroimaging task details, controls, and neural activations). If information was missing from a study, it was decided that the first author would contact the relevant corresponding author, requesting the information. After two weeks if no response was received, the information was left as missing. No data in this review was acquired in this way.

### Quality Assessment

Methodological quality of included studies was measured using a modified version of the Downs and Black Quality Assessment checklist (Downs & Black, 1998). Quality assessment was completed independently by the first and second author, then results compared to ensure consistency. As some studies are single-case, certain questions are not applicable, therefore percentage scores were chosen to assess quality of papers. Quality levels were as follows: *Excellent Quality* ≥ 75%, *Moderate Quality* 50% to 74%, *Low Quality* 25% to 49%, and *Poor quality* ≤ 25%.

### Synthesis Methods

Outcome measures and statistical analyses implemented in included studies are highly varied, therefore, a meta-analysis was not performed to synthesise findings. The variability discovered was beyond the limits described in the literature (Ioannidis et al., 2008). For synthesis of findings, extracted information was used to group results into categories based on the methodologies implemented. Categories were as follows: behavioural, structural magnetic resonance imaging (MRI), task-based functional magnetic resonance imaging (fMRI), and resting-state fMRI results. Studies appear in multiple categories when several methodologies were used. Once categorised, results were presented in table form using Microsoft Word for formatting, alongside written descriptions in the result sections.

## Results

### Study Selection

**Fig. 1:**
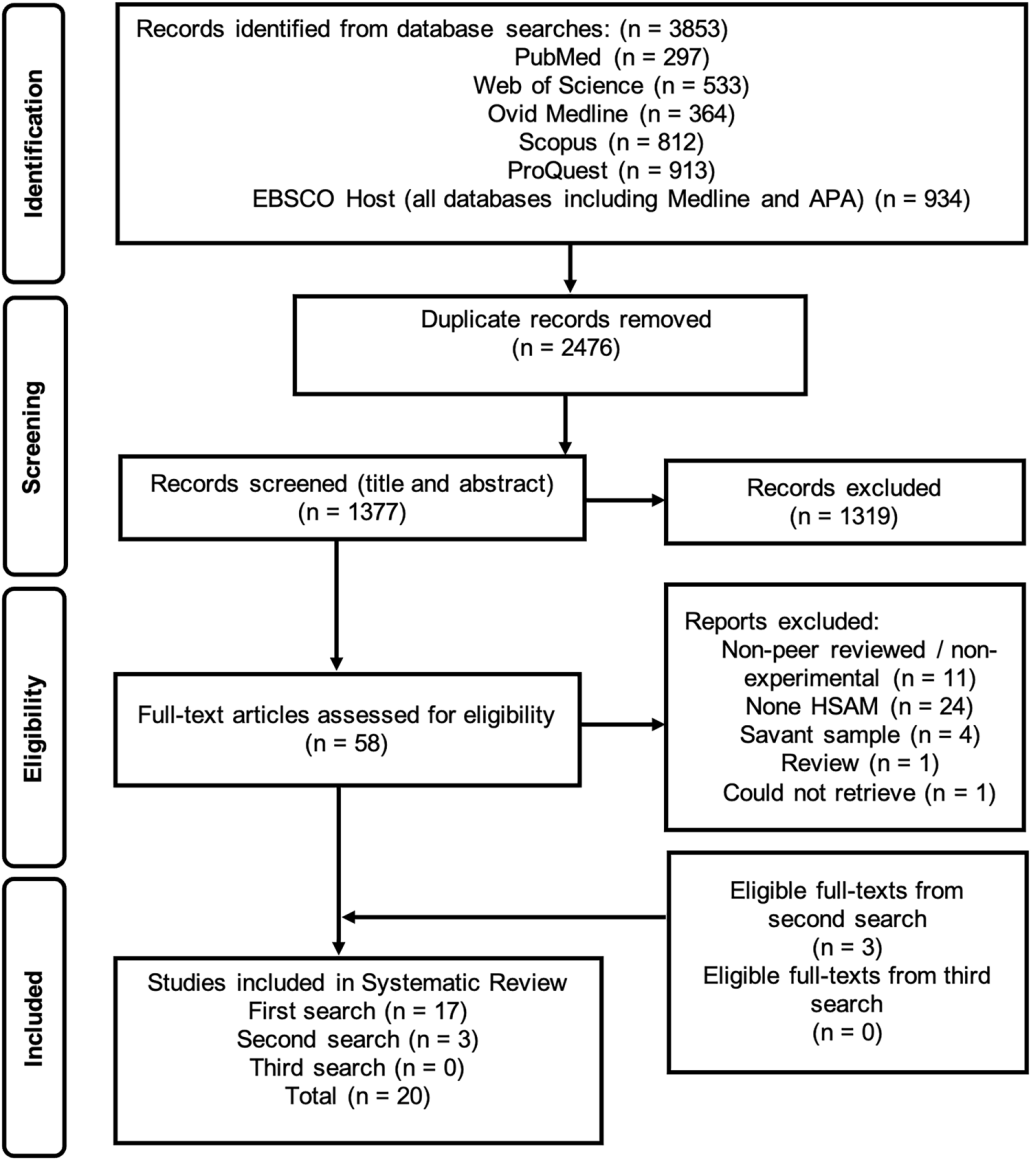
Study selection process (PRISMA flow chart)

The initial search identified 3853 results. 2476 duplicate records were manually removed, and the remaining 1377 records were independently screened based on their title and abstract. Next, full-texts of fifty-eight records were independently assessed for eligibility and seventeen articles were included. An additional two searches were run approximately six months and one year later, following the same study selection process, and three additional papers were identified. Twenty full-text articles are included in this review. The PRISMA flow diagram illustrating the initial study selection process can be seen in Fig. 1. Several studies appeared to meet inclusion criteria by reporting participants with enhanced cognitive abilities (e.g., Cook Maher et al., 2017; De Marco et al., 2015; Mella et al., 2021). However, full-text assessments revealed that the exceptional traits described were distinct from ABM, and therefore, they were excluded.

### Study Characteristics and HSAM Participant Information

Table 1 displays study characteristics, including geographical location of researchers, main objectives, and methodologies used. The United States of America (USA) is the country with the most publications on HSAM; thirteen studies received contributions from institutions located there (Ally et al., 2013; Brandt & Bakker, 2018; Daviddi et al., 2022b; Frithsen et al., 2018; LePort et al., 2012, 2016, 2017; Levine et al., 2019, 2021; Parker et al., 2006; Patihis, 2015; Patihis et al., 2013; Santangelo et al., 2018). Eight of the twenty included studies are single case (Ally et al., 2013; Brandt & Bakker, 2018; De Marco et al., 2021; Ford et al., 2022; Gibson et al., 2022; Mazzoni et al., 2019; Parker et al., 2006; Santangelo et al., 2021). Case abbreviations (e.g., ‘RS’) are in Table 1 and will be used throughout this review.

**Table 1:** Characteristics of studies included in this systematic review, including the main methodologies utilised

Ref	Location	Study type	Study design	Study aims and objectives
Parker et al. (2006)	USA	Single case	Behaviour assessment	Outline the first modern case of exceptional ABM, explore abilities and clinical profile of *AJ*
Ally et al. (2013)	USA	Single case	Behaviour assessment Resting-state fMRI Structural MRI	Explore the cognitive, intellectual, and neural underpinnings of *HK*
Brandt and Bakker (2018)	USA	Single case	Behaviour task Resting-state fMRI Structural MRI	Investigate a HSAM case (*MM*) with a broader range of skills than others (i.e., an encyclopaedic knowledge)
Mazzoni et al. (2019)	Italy, UK	Single case	Behaviour assessment Task-based fMRI Structural MRI	Measure brain activations of *BB* during a retrieval task using dates, explore if OCD is a prerequisite for HSAM
De Marco et al. (2021)	UK, Italy	Single case	Resting-state fMRI Structural MRI	Investigate resting-state connectivity of *BB* vs. controls
Santangelo et al. (2021)	Italy	Single case	Behaviour assessment Task-based fMRI Structural MRI	Explore the relationship between aging and memory in HSAM, Present the oldest HSAM participant (*GC*) identified by researchers
Gibson et al. (2022)	Australia	Single case	Behaviour assessment Structural MRI	Investigate future thinking abilities in *RS*
Ford et al. (2022)	Australia	Single case	Behaviour assessment Structural MRI	Explore how memory representations are structured in *RS* using novel tasks with objectively verifiable content (e.g., day of the week)
LePort et al. (2012)	USA	Group	Behaviour assessment Structural MRI	Identify similarities and differences in behavioural performance and brain structure in HSAM individuals vs. controls
Patihis et al. (2013)	USA	Group	Behaviour assessment	Explore false memories in HSAM populations
Patihis (2015)	USA	Group	Behaviour assessment	Identify similarities and differences in untested domains in HSAM individuals vs. controls
LePort et al. (2016)	USA	Group	Behaviour assessment	Investigate similarities and differences in quality and quantity of ABM over time (HSAM vs. controls), explore contribution of OCD to HSAM
LePort et al. (2016)	USA	Group	Behaviour assessment	Identify similarities and differences in behavioural performance in HSAM individuals vs. controls
Frithsen, et al. (2018)	USA	Group	Behaviour assessment	Explore response bias and recollection performance in HSAM vs. controls
Levine et al. (2019)	USA	Group	Behaviour assessment	Investigate accuracy at predicting or remembering emotional responses to events (HSAM vs. controls) *aims-objectives relevant to this review
Levine et al. (2021) Study 1 only	USA, Ireland, Italy, UK	Group	Behaviour assessment	Explore accuracy at remembering feelings and facts of a political event (HSAM vs. controls) *aims-objectives relevant to this review
Santangelo et al. (2018)	Italy, USA	Group	Task-based fMRI	Identify neural activations during retrieval of ABM and semantic memories (HSAM vs. controls)
Santangelo et al. (2020)	Italy, Sweden	Group	Task-based fMRI	Reanalyse data (Santangelo et al., 2018) using newer analysis methods, explore if select brain areas distinguish older and newer memories
Daviddi et al. (2022a)	Italy	Group	Resting-state fMRI Structural MRI	Explore resting-state functional connectivity of the hippocampus and other brain areas (HSAM vs. controls)
Daviddi et al. (2022b)	Italy, USA	Group	Behaviour assessment	Explore creative thinking in HSAM (HSAM vs. controls)

HSAM participant characteristics (not controls) are presented in Table 2. Participants’ age varied between nineteen (LePort et al., 2016) and 80 years old (Santangelo et al., 2021). More males than females have been reported with HSAM, although exact numbers of each sex cannot be determined due to lack of clarity about participants appearing in multiple studies. Right handedness was more commonly reported than left-handedness in HSAM (Brandt & Bakker, 2018; De Marco et al., 2021; Ford et al., 2022; Gibson et al., 2022; LePort et al., 2012; Mazzoni et al., 2019; Parker et al., 2006; Patihis, 2015). Parker et al. (2006) described anomalous hand dominance in AJ; despite stating she was right-handed, photographs showed her playing with her left-hand during childhood and she worked from left-to-right on tasks normally performed in reverse by right-handed participants. Of the studies that reported current occupation, no HSAM participants were consistently employed. Reasons for this varied; MM was occupationally disabled (Brandt & Bakker, 2018), AJ was a mother (Parker et al., 2006), and BB was a student (De Marco et al., 2021; Mazzoni et al., 2019).^1^

**Table 2:** Details of the HSAM participants included in each study

Ref	HSAM sample demographics				Participant screening		
	Size	Sex	Age	Hand	HSAM screen	Clinical screen tool	Clinical profile and results
Parker et al. (2006)	1	F = 1	*Born 1965*	1 = R	Cognitive battery	Medical history Interviews	Anxiety medication, previous depressive periods, phobias, (e.g., specific smells), used diaries, needs order
Ally et al. (2013)	1	M = 1	20	NS	Cognitive battery	Medical history	Completely blind
Brandt and Bakker (2018)	1	M = 1	63	1 = R	Cognitive battery	Medical history Interviews PAI, AQ, E, AAA	Major depressive disorder, prominent anxiety. treated with medication and psychotherapy, PAI profile normal, did not meet Asperger’s-autism criteria
Mazzoni et al. (2019)	1	M = 1	20	R	HMSQ	HMSQ	Healthy, no signs of OCD or autism
De Marco et al. (2021)	1	M = 1	20	R	See Mazzoni et al. (2019)	See Mazzoni et al. (2019)	See Mazzoni et al. (2019)
Santangelo et al. (2021)	1	M = 1	75 & 80	NS	PEQ RDQ	MMSE, PAI	Cognitive capabilities normal range, scored in upper quartile MMSE PAI found no clinical symptoms, including OCD
Gibson et al. (2022)	1	F = 1	31 *Most tests at 26	R	PEQ RDQ	Medical history HADS	Mid-functioning ASD, PTSD, depression, GAD, OCD, HADS showed higher depressive symptoms vs. controls, but anxiety levels comparable
Ford et al. (2022)	1	F = 1	31	R	See Gibson et al. (2022)	See Gibson et al. (2022)	See Gibson et al. (2022)
LePort et al. (2012)	11	M = 7 F = 4	M = 43 R = 27–60	R = 6 L = 3 A = 2	PEQ 10DQ	LOI-SF, Behaviour Questionnaire, BDI-II	mean OCD score significantly higher HSAM vs. controls, 3/11 kept diaries, 9/11 report hoarding, HSAM group in minimal depression range
Patihis et al. (2013)	20	See Patihis (2015)	M = 38.6 R = 21–62	See Patihis (2015)	PEQ 10DQ	See Patihis (2015)	See Patihis (2015)
Patihis (2015)	20	M = 15 F = 5	See Patihis et al. (2013)	Overall right-hand preference	PEQ 10DQ	SSP	Psychic trait anxiety significantly higher in HSAM sample vs. control, somatic trait anxiety scores did not differ
LePort et al. (2016)	30	M = 24 F = 6	M = 39 R = 19–68	NS	PEQ 10DQ	LOI	Mean OCD score significantly higher HSAM vs. controls
LePort et al. (2017)	20	M = 13 F = 7	M = 37.5 R = 20–53	NS	PEQ 10DQ	See LePort et al. (2012) for details of some participants	See LePort et al. (2012) for details of some participants
Frithsen et al. (2018)	15	M = 12 F = 3	M = 38 R = 21–64	NS	PEQ 10DQ	NS	NS
Levine et al. (2019)	33	See Levine et al. (2021)	See Levine et al. (2021)	NS	PEQ 10DQ	NS	NS
Levine et al. (2021) Study 1	33	M = 25 F = 7	M = 42.19 R = NS	NS	PEQ 10DQ	NS	NS
Santangelo et al. (2018)	8	M = 5 F = 3	M = 32.5 R 24–37	NS	PEQ 10DQ	PAI	HSAM participants in 92nd percentile for obsessive-compulsive symptoms
Santangelo et al. (2020)	8	M = 5 F = 3	M = 32.5 R = 24–37	NS	PEQ 10DQ	See Santangelo et al. (2018)	See Santangelo et al. (2018)
Daviddi et al. (2022a)	8	M = 5 F = 3	M = 32.5 R = 24–37	NS	PEQ 10DQ	See Santangelo et al. (2018)	See Santangelo et al. (2018)
Daviddi et al. (2022b)	14	M = 9 F = 5	M = 35.07 R = 20–47	NS	PEQ RDQ	NS	NS

*PAI* Personality Assessment Inventory, *AQ* Autism Quotient, *EQ* Empathy Questionnaire, *AAA* Adult Asperger Assessment, *MMSE* Mini-Mental State Examination, *LOI-SF* Leyton Obsessional Inventory Short Form, *SSP* Swedish University Scale of Personality, *HADS* Hospital Anxiety and Depression Scale, *ASD* Autism Spectrum Disorder, *PTSD* Post-Traumatic Stress Disorder, *GAD* Generalised Anxiety Disorder, *OCD* Obsessive Compulsive Disorder, *BDI-II* Beck’s Depression Inventory II, *LOI* Leyton Obsessional Inventory, *HMSQ* Hull Memory Screening Questionnaire, *PEQ* Public Events Quiz, *RDQ* Random Dates Quiz, *10DQ* 10 Dates Quiz

The most frequently used HSAM screening tools were the Public Events Quiz (PEQ), followed by the Random Dates Quiz (synonymously 10 Dates Quiz). Two studies used the Hull Memory Screening Questionnaire (HMSQ) (De Marco et al., 2021; Mazzoni et al., 2019). Three studies did not explicitly state which tasks were used for HSAM screening; however, the included HSAM participants completed in depth neuropsychological assessments (Ally et al., 2013; Brandt & Bakker, 2018; Parker et al., 2006).

Clinical profiles of HSAM participants are highly heterogenous. Many participants have obsessive compulsive tendencies (Ford et al., 2022; Gibson et al., 2022; LePort et al., 2012, 2016; Parker et al., 2006; Santangelo et al., 2018). More specifically, LePort et al. (2012) used the short form version of the Leyton Obsessional Inventory (LOI) to measure obsessional tendencies. 81.18% of their HSAM sample reported traits such as hoarding items or being avoidant of germs. Later, LePort et al. (2016) administered the long-form version of the LOI to produce a symptom score. The symptom scores of the HSAM sample (M = 31.75, SD = 11.02) were normalised using *z*-scores to an OCD population’s normative data (M = 33.3, SD = 7.7). HSAM scores were found to be indistinguishable from the OCD population. Personality Assessment Inventory data obtained by Santangelo et al. (2018) showed that for the “obsessive–compulsive” subscale, the overall mean HSAM score was in the 92nd percentile relating to obsessive and compulsive symptoms. Elevated psychological trait anxiety scores (Patihis, 2015), and presence of anxiety conditions (Brandt & Bakker, 2018; Gibson et al., 2022; Parker et al., 2006) have also been reported. However, BB (De Marco et al., 2021; Mazzoni et al., 2019) and GC (Santangelo et al., 2021) showed no clinical traits. Group studies have shown that HSAM participants are not within the clinical depression range (LePort et al., 2012), but single-cases have reported current depression diagnoses (Brandt & Bakker, 2018; Ford et al., 2022; Gibson et al., 2022) and previous depressive periods (Parker et al., 2006).

### Quality Assessment

Risk of bias was assessed using a modified version of the Downs and Black Quality Assessment checklist (Downs & Black, 1998). Results are presented in Table 3. Fourteen studies were considered “excellent quality” and six “moderate quality”.

**Table 3:** Methodological quality assessment results using a modified version of the Downs and Black Quality Assessment checklist

Reference	Reporting							External validity		Internal validity						Quality
										**Bias**					**Confounding**	
	**Q1** **(2)**	**Q2**	**Q3** **(2)**	**Q5** **(2)**	**Q6**	**Q7**	**Q10**	**Q11**	**Q12** **(2)**	**Q16**	**Q17**	**Q18**	**Q19**	**Q20**	**Q25** **(2)**	**(%)**
Parker et al. (2006)	1	1	2	N/A	1	1	N/A	1	0	N/A	N/A	N/A	1	1	N/A	81.8%
Ally et al. (2013)	1	1	1	1	1	1	1	1	0	1	1	1	1	1	1	73.7%
Brandt and Bakker (2018)	2	N/A	2	1	1	1	0	1	2	1	0	1	1	1	0	77.8%
Mazzoni et al. (2019)	2	1	2	1	1	1	1	1	1	1	1	1	1	1	0	84.40%
De Marco et al. (2021)	2	1	2	2	1	1	1	1	0	1	0	1	1	1	0	78.90%
Santangelo et al. (2021)	1	1	1	N/A	1	1	1	1	1	1	N/A	1	N/A	1	N/A	84.65%
Gibson et al. (2022)	2	1	2	1	1	1	1	1	N/A	1	0	1	1	1	0	82.30%
Ford et al. (2022)	2	1	2	1	1	1	1	1	N/A	1	0	1	1	1	0	82.30%
LePort et al. (2012)	1	1	2	1	1	1	1	1	1	1	1	1	1	1	1	84.20%
Patihis et al. (2013)	2	1	1	1	1	1	1	1	1	1	1	1	1	1	1	84.20%
Patihis (2015)	2	1	2	1	1	1	1	1	0	1	0	1	1	1	1	78.90%
LePort et al. (2016)	1	1	2	1	1	1	1	1	1	1	1	1	1	1	1	84.20%
LePort et al. (2017)	2	1	2	2	1	1	1	1	0	1	1	1	1	1	0	84.20%
Frithsen et al. (2018)	2	0	1	2	1	1	1	1	0	1	1	1	1	1	0	73.70%
Levine et al. (2019)	2	1	1	0	1	1	1	1	0	1	1	1	0	1	0	63.20%
Levine et al. (2021)	2	1	1	0	1	1	1	1	0	1	1	1	0	1	0	63.20%
Santangelo et al. (2018)	2	1	1	1	1	1	1	1	1	1	1	1	1	1	1	84.20%
Santangelo et al. (2020)	2	1	1	1	1	1	1	1	0	1	1	1	1	1	0	73.70%
Daviddi et al. (2022a)	2	1	1	1	1	1	1	1	0	1	1	1	1	1	0	73.70%
Daviddi et al. (2022b)	2	1	2	2	1	1	1	1	0	1	1	1	1	1	0	84.20%

### Main Behavioural Results

Main behavioural results are summarised below (see Supplementary Materials for a more detailed list). All twenty HSAM studies reported enhanced ABM performance. When dates were retrieval cues, test–retest reliability was perfect (Ally et al., 2013; Parker et al., 2006) and verifiable detail accuracy was exceptional (98% accuracy) (Mazzoni et al., 2019). GC passed HSAM assessment at 75 and 80 years old (Santangelo et al., 2021). In fact, PEQ performance improved (approximately 12%) between timepoints and memories remained high in episodic details. In ad hoc tasks designed to assess semantic and ABM, RS performed significantly better than matched controls (Ford et al., 2022). Gibson et al. (2022) hypothesised enhanced past ABM may coincide with enhanced future thinking (i.e., a capacity to disengage from the present and mentally project oneself into the future to imagine hypothetical scenarios) (see D’Argembeau et al., 2010). When single words were used as cues to simulate a future autobiographical event (Adapted Autobiographical Interview), RS described more detailed events than controls. However, in future thinking tasks not related to one’s own experiences (Narrative Scene Construction – Cinderella and Cookie Theft), performance was comparable to controls, and RS repeated herself more.

HSAM individuals were found to have an enhanced performance for some tasks that did not measure ABM. On a measure of associative memory, LePort et al. (2012) found that HSAM individuals had superior Names to Faces task performance, compared with controls. This finding was confirmed later by significantly higher HSAM Face-Name-Occupations Task scores (LePort et al., 2017). Despite these results, on other tasks which involve aspects of associative memory (e.g., the three-phase story), researchers did not find that the HSAM group were superior. Enhanced or excellent performance was found for olfactory functioning (Parker et al., 2006), celebrity face recognition (Brandt & Bakker, 2018), word recognition (Parker et al., 2006), and narrative abilities as measured by the Script Generation Task (LePort et al., 2017). “Absorption” and “fantasy proneness” personality traits, measured by the Tellegen Absorption Scale and Creative Experience Questionnaire, respectively (Patihis, 2015), were significantly higher than controls.

On some measures of ABM performance, HSAM and controls were comparable. During the Meta Test (i.e., to quantify retrieval of the whole testing experience), the experimenter asked participants questions about their life (e.g., “How was your weekend?”). Participants provided responses and the experimenter also offered a story in return (e.g., a story about seeing a gun on campus) (LePort et al., 2017). One week and one month later, participants were tested on their memory of these responses. Whilst HSAM participants excelled at the personal recollections, their recall for the experimenters’ anecdotes was entirely analogous with the control population. In a Dates task, HSAM participants provided higher quantity and quality of memories for remote time periods (1 month, 1 year, and 10 years from memory testing) (LePort et al., 2016) but comparable responses to controls for dates 1 week from testing. When completing the Autobiographical Interview, RS was comparable to controls during free recall of specific events from time periods of her life (e.g., adolescence) and provided fewer external elements (i.e., semantic details not specific to events) during a single event from Early Adulthood (Gibson et al., 2022).

LePort et al. (2017) administered the Three Phase Story to explore memory retrieval for a story that induced negative emotional arousal. When exposed to emotional stimuli HSAM participants did not recall more than controls. Similarly, HSAM participants were no better than controls at predicting how emotional they would feel at an upcoming political election (Levine et al., 2019) or remembering their emotions three weeks (Levine et al., 2019), or six months post-election (Levine et al., 2021). HSAM participants reported feeling high arousal emotions as frequently as controls (Patihis, 2015). Other cognitive domains that were associated with performance that was not statistically different from controls or normative scores in HSAM included verbal (LePort et al., 2012, 2017; Parker et al., 2006), prospective (Brandt & Bakker, 2018; Gibson et al., 2022), and semantic memory (Parker et al., 2006). Language (Gibson et al., 2022; Mazzoni et al., 2019; Parker et al., 2006), mental imagery (LePort et al., 2017), and creative (Daviddi et al., 2022b) or critical (Patihis, 2015) thinking were also not statistically different to controls or normative scores. Questionnaires indicated sleep was not altered in HSAM (Patihis, 2015). These results indicate that for people with HSAM their cognitive skills for non-autobiographical tasks are well within the range of normality. These conclusions require accepting the null hypothesis; it would therefore be highly beneficial to calculate the Bayes factor. Due to lack of relevant information in the published articles this was not feasible.

HSAM individuals’ intelligence was generally in the normal range (Ally et al., 2013; Brandt & Bakker, 2018; Gibson et al., 2022; Parker et al., 2006; Patihis, 2015). BB demonstrated overall intelligence in the 90th percentile (Mazzoni et al., 2019). Five studies administered Digit Span tasks to assess attention and working memory in HSAM; three found average results (Daviddi et al., 2022b; Gibson et al., 2022; LePort et al., 2012), and the remaining reported scores better than reference controls (Mazzoni et al., 2019; Parker et al., 2006). Visual memory and visuospatial abilities varied between studies; above average performance was found in the Visual Memory Index (Parker et al., 2006) and Visual Reproduction subtests of the Wechsler Memory Scale—Revised (Mazzoni et al., 2019). Conversely, performance was average for tests such as Visual Reproduction (LePort et al., 2012), Visual Patterns, and the Progressive Silhouettes task (LePort et al., 2017). When administering the Wechsler Memory Scale (WMS) to assess memory performance, two studies found average overall performance (Ally et al., 2013; Brandt & Bakker, 2018) and one study (LePort et al., 2012) found average performance for Logical Memory (recognition). Conversely, AJ scored near maximum for the WMS General Memory Index (Parker et al., 2006) and HSAM participants were superior at Logical Memory (free recall) (LePort et al., 2012). HSAM individuals were as susceptible as controls to false memories in the Deese, Roediger, and McDermott (DRM) task and on the non-existent news footage paradigm and had slightly more overall false memories during the Misinformation task (Patihis et al., 2013).

Areas of cognitive weakness also varied. AJ showed impairments in tasks assessing aspects of executive functioning, including shifting measured by the Wisconsin Card Sorting Test (Parker et al., 2006). Other studies, which used tasks like the classic Stroop, found executive functioning elements (e.g., interference, initiation, and inhibition) to be intact (Gibson et al., 2022; Mazzoni et al., 2019). A motor speed impairment was reported for AJ (Parker et al., 2006), but scores were average for RS (Gibson et al., 2022). HSAM individuals have lower flexible thinking scores, particularly relating to “tolerance for ambiguity” (Patihis, 2015). Cognitive and affective empathy was reported as normal in HSAM (Patihis, 2015), assessed with the Empathy Questionnaire and Basic Empathy Scale scores, respectively. RS was deficient in her ability to “comprehend emotions of others” (Gibson et al., 2022). HSAM individuals had a lower response bias criterion, indicating a more liberal tendency to report items as previously seen (Frithsen et al., 2018).

### Structural Neuroimaging Findings

Main MRI findings and details of controls are summarised in Table 4. Four studies (Ally et al., 2013; Brandt & Bakker, 2018; LePort et al., 2012; Mazzoni et al., 2019) found anatomical differences spanning both hemispheres in HSAM participants. In the left hemisphere, MRI data from BB (Mazzoni et al., 2019) revealed significantly bigger grey-matter volumes compared with controls from an occipitotemporal cluster extending to the posterior hippocampus. Brandt and Bakker (2018) found an increase in the bilateral temporopolar cortex total volume in MM, and Ally et al. (2013) reported a bigger subcortical volume of the right amygdala in HK. Diffusion Tensor Imaging analysis performed by LePort et al. (2012) revealed increased anisotropy (i.e., indicative of better signal conduction) bilaterally in the forceps major, parahippocampal gyrus and intraparietal sulcus in HSAM participants (vs. controls). This was accompanied by increased anisotropy in the left uncinate fasciculus and right lingual gyrus. Tensor based morphometry (TBM) analysis showed bigger volumes in the left posterior insula, whilst voxel-based morphometry-grey matter (VBM-GM) analysis revealed bigger size of right hemisphere structures (e.g., posterior pallidum) in HSAM participants.

**Table 4:** Main structural neuroimaging results from studies finding neuroanatomical differences in HSAM participants

Ref	Controls	Measure	↑ HSAM sample vs. controls	↓ HSAM sample vs. controls
Ally et al. (2013)	30	Volume	Amygdala (right)	Thalamus, caudate, putamen, pallidum, hippocampus (bilateral), amygdala (left), total tissue volume
Brandt and Bakker (2018)	5	Volume	Temporal polar cortex (bilateral)	Perirhinal cortex (right), “*possibly”* entorhinal cortex (left)
Mazzoni et al. (2019)	10	Grey-matter volume	Lingual gyrus, middle temporal gyrus, cuneus, insula, posterior cingulate, caudate tail, superior temporal gyrus, putamen, fusiform gyrus (left)	-
LePort et al. (2012)	19	a. DTI-FA b. VBM-GM c. VBM-WM d. TBM	a. Uncinate fasciculus (left), forceps major, parahippocampal gyrus, intraparietal sulcus (bilateral), lingual gyrus (right) b. Anterior putamen & caudate surrounding anterior limb of internal capsule, posterior pallidum (right) c. - d. Posterior insula (left)	a. - b. Anterior and middle temporal gyrus, interparietal sulcus c. Anterior putamen & caudate surrounding anterior limb of internal capsule, anterior and middle temporal gyrus (bilateral), posterior pallidum, lingual gyrus (right) d. Anterior putamen & caudate surrounding anterior limb of internal capsule, anterior and middle temporal gyrus (left), lingual gyrus (right)

*DTI-FA* Diffusion Tensor Imaging-Fractional Anisotropy, *VBM-GM* voxel-based morphometry-grey matter, *VBM-WM* voxel-based morphometry-white matter, *TBM* tensor-based morphometry

Conversely, in MM, the right perirhinal cortex was smaller in total size (Brandt & Bakker, 2018) compared with control data. Ally et al. (2013) found significant reductions in size of bilateral subcortical structures (i.e., thalamus, caudate, putamen, pallidum, hippocampus, and the left amygdala) for HK. Lower grey and white matter concentrations for HSAM participants were found when VBM-GM and voxel-based morphometry-white matter analysis was performed to assess the anterior and middle temporal gyrus, bilaterally (LePort et al., 2012). In the same study, reduced grey matter relative concentrations were discovered by VBM-GM in the vicinity of the bilateral intraparietal sulcus.

The remaining structural neuroimaging studies found no significant structural differences (Ford et al., 2022; Gibson et al., 2022; Santangelo et al., 2021). Two studies (Daviddi et al., 2022a; De Marco et al., 2021) obtained MRI data to confirm absence of neurostructural abnormalities that may explain between group resting-state connectivity differences and found no significant anatomical differences.

Overall, the statistical effects observed in the included neuroanatomical data indicate an involvement of subcortical nuclei and mediotemporal-limbic and temporo-occipital regions in HSAM. Moreover, the collated evidence shows an asymmetric trend suggesting that structural alterations may be linked to functional hemispheric specialisation.

### Resting-State Functional Connectivity Results

Results from studies reporting resting-state fMRI data are presented in Table 5. All studies (Ally et al., 2013; Brandt & Bakker, 2018; Daviddi et al., 2022a; De Marco et al., 2021) reported between group differences in resting-state functional connectivity, and each investigated region-to-region connectivity. One study explored within network functional connectivity (Ally et al., 2013) and another analysed large scale brain networks and used graph theory approaches to process data (De Marco et al., 2021).

**Table 5:** Main resting-state functional connectivity findings for included HSAM studies

Ref	Controls	Side	Region of interest or seed region	Finding	Side	Connected brain area
Ally et al. (2013)	10	h. R	*Within-network functional connectivity:* a. Posterior cingulate-ventral precuneus network b. Inferior parietal network hub c. Cuneus network hub d. Postcentral network hub e. Cerebellum network hub f. Thalamus network hub g. Amygdala network hub *Between area functional connectivity:* h. Amygdala	a. Decreased activity versus controls b. No difference c. No difference d. Increased activity e. No difference f. Increased activity g. No difference h. Increased activity with:	h. R	h. Hippocampus
Brandt and Bakker (2018)	5	a. L b. L	*Between area functional connectivity:* a. Hippocampus b. Hippocampus	a. Increased activity with: b. Decreased activity with:	a. L a. B b. L b. B	a. Inferior prefrontal cortex, inferior frontal cortex–pars opercularis, premotor cortex, retrosplenial cingulate cortex a. Dorsolateral prefrontal cortex b. Posterior entorhinal cortex b. Perirhinal cortex
De Marco et al. (2021)	16	a. B a. B b. L b. L b. B c. L d. R e. R f. L g. R g. R g. R	*Large-scale brain networks:* a. Superior temporal gyrus a. Inferior parietal lobule b. Inferior temporal gyrus b. Superior temporal gyrus b. Uncus *Region of interest to region of interest functional connectivity:* c. Lingual gyrus d. Orbitofrontal cortex e. Heschl’s gyrus Graph theory: f. Globus pallidus g. Temporal pole g. Orbitofrontal cortex g. Cerebellar lobule IX	a. Default-mode network regions more active in BB b. ABM network areas more active in BB than controls c. Increased activity with: d. Increased activity with: e. Increased activity with: f. BB reduced local efficiency & clustering coefficient g. Betweenness centrality higher in BB than controls	c. R d. R e. L	c. Heschl’s gyrus d. Cerebellar lobule IX e. Planum temporale
Daviddi et al. (2022a)	21	a. R b. L c. L d. R e. R	*Between area functional connectivity:* a. Anterior hippocampus b. Anterior hippocampus c. Posterior hippocampus d. Anterior hippocampus e. Posterior hippocampus	a. Decreased activity with: b. Decreased activity with: c. Decreased activity with: d. Increased activity with: e. Increased activity with:	a. B b. L b. R c. L d. L e. B	a. Insula, temporoparietal junction, anterior cingulate cortex b. Middle frontal gyrus, supramarginal gyrus, inferior precentral gyrus b. Superior frontal gyrus c. Inferior frontal gyrus, middle cingulate cortex d. Fusiform gyrus e. Inferior temporal gyri

Three studies found greater hippocampus connectivity with other brain areas. Brandt and Bakker (2018) found the left hippocampus had greater connectivity with left hemisphere regions (inferior prefrontal cortex, inferior frontal gyrus–pars opercularis, premotor cortex, and retrosplenial cingulate cortex) and the bilateral dorsolateral prefrontal cortex, in HSAM vs. controls. Ally et al. (2013) found the right amygdala had increased functional connectivity with the right hippocampus, and Daviddi et al. (2022a) reported the right anterior hippocampus and right posterior hippocampus had greater connectivity with the left fusiform gyrus and bilateral inferior temporal gyrus, respectively.

Contrastingly, weaker hippocampus resting-state connectivity was found in HSAM participants vs. controls. Less left hippocampus connectivity was reported with the left posterior entorhinal cortex and bilateral perirhinal cortex (Brandt & Bakker, 2018). Weaker anterior left hippocampus connectivity was found with the left middle frontal gyrus, supramarginal gyrus, inferior precentral gyrus, and right superior frontal gyrus (Daviddi et al., 2022a). The posterior left hippocampus had less connectivity with the left inferior frontal gyrus and middle cingulate cortex. The right anterior hippocampus had weaker functional connectivity bilaterally with the insula, temporoparietal junction and anterior cingulate cortex (Daviddi et al., 2022a). Lower levels of resting-state functional connectivity were found in the posterior cingulate and ventral precuneus network (Ally et al., 2013).

Resting-state connectivity related to the cerebellum yields significant between-study variability in HSAM vs. controls. Ally et al. (2013) found no difference between within-network cerebellum connectivity in the seed-based cerebellar connectivity network analysis. Contrastingly, De Marco et al. (2021) found the right orbitofrontal cortex had more connectivity with the right cerebellar lobule IX. Graph theory analysis in the same study found the right cerebellar lobule IX had higher levels of betweenness centrality in BB than controls. Higher levels of betweenness centrality (a measure of pathway-related relevance assumed by a region) were also reported in the right temporal pole and right orbitofrontal cortex. The left globus pallidus was found to have lower levels of local efficiency and clustering coefficient (i.e., two metrics indicative of local integration).

Stronger connectivity was found in patterns of inter-regional connectivity (e.g., between left lingual gyrus and right Heschl’s gyrus) (De Marco et al., 2021) and in the postcentral and thalamic networks (Ally et al., 2013) of HSAM participants. Large-scale brain network analysis revealed default-mode network regions bilaterally (superior temporal gyrus and inferior parietal lobule) and ABM network areas (e.g., left superior and inferior temporal gyrus) were more significantly expressed in BB than controls (De Marco et al., 2021).

To summarise, these results indicate significant alterations to mediotemporal, limbic, and prefrontal neural pathways that typically support high-order cognitive abilities such as language or speech processing and memory. As the methodologies deployed in these studies are quite diverse, there is considerable variability in the emerging pattern of findings. It should also be noted that, whilst the differences observed in this section could be linked to superior memory; they could also be associated with idiosyncrasies of neurofunctional architecture.

### Task-Based fMRI Results

Table 6 presents details of the fMRI tasks that have been used to explore HSAM retrieval and provides the related main behavioural results. In two studies ABM cues prompted participants to remember the “first” or “last time” they experienced an event (Santangelo et al., 2018, 2020). In two studies, dates from the participants life were used as memory cues (Mazzoni et al., 2019; Santangelo et al., 2021). Participants reported when a memory was initially accessed and, again, when they “elaborated” (Mazzoni et al., 2019) or “relived” (Santangelo et al., 2018, 2020, 2021) this memory. Table 7 presents the main neural activations recorded by task-based fMRI studies.

**Table 6:** Details of task-based fMRI studies and main behavioural results

Ref	Controls	Task details	Stimuli used	Main fMRI behaviour results
Santangelo et al. (2018)	21	Cues provided and participants report access and reliving time of related memory	Autobiographical: “First or last time you did…” Semantic: Such as lists of vegetables	HSAM group accessed ABM’s faster HSAM group provided more details for ABM’s No difference HSAM versus controls semantic memory RT No difference HSAM versus controls for ABM self-report ratings of emotional intensity or reliving HSAM group: no correlation between faster RT or number of ABM details with obsessive–compulsive trait scores
Mazzoni et al. (2019)	0	Cues given and BB reports access and elaboration time of related personal memory	Autobiographical: Dates from BB’s lifetime 2 sets of dates tested 3 months earlier: Set 1 = 50 “Yes” dates BB reported a memory for Set 2 = 50 “No” dates BB reported no recollection of	BB reported a memory for 43/50 “Yes” dates and 15/50 “No” dates Mean access time: “Yes” dates = 1816 ms (SD = 1305 ms) Mean access time: “No” dates = 1952 ms (SD = 1253 ms) Mean elaboration time: “Yes” dates = 11,725 ms (SD = 3750 ms) Mean elaboration time: “No” dates = 16,196 ms (SD = 3694 ms)
Santangelo et al. (2021)	0	Cues given and GC reports access and reliving time of related personal memory Emotional valence and reliving quality ratings made for each ABM	Autobiographical: Dates from GC’s lifetime 18 dates	GC retrieved a memory for 15/18 dates (83%) 15/15 dates GC provided detailed descriptions post-scanner 15/15 dates GC provided a verifiable event ± 1 month of the specific date and day of the week

*RT* response time

**Table 7:** Main neural activation results obtained from task-based fMRI studies

Ref	Measure	Side	Brain area
Santangelo et al. (2018)	a. Increased activity HSAM vs. controls (overall retrieval) b. Areas involved selectively with HSAM access c. Areas contributing more to older (vs. new) HSAM ABMs d. Increased activity controls (overall retrieval) e. Left ventromedial prefrontal cortex functionally couples with: f. Left dorsomedial prefrontal cortex functionally couples with: g. Left temporoparietal junction functional couples with:	a. B a. R a. L b. L c. L d. L d. R e. L e. B e. R f. L f. B f. R g. B g. L	a. Angular gyrus, ventromedial prefrontal cortex a. Dorsolateral prefrontal cortex, insula a. Temporoparietal junction b. Temporoparietal junction, ventromedial prefrontal cortex, dorsomedial prefrontal cortex c. Ventromedial prefrontal cortex d. Middle occipital gyrus, posterior cingulate cortex d. Superior frontal sulcus e. Hippocampus, temporoparietal junction, supramarginal gyrus, superior temporal cortex e. Subcentral gyrus, rostral anterior cingulate cortex e. Post-central gyrus f. Precentral gyrus, superior frontal gyrus, precuneus, superior parietal lobule f. Ventromedial prefrontal cortex, intraparietal sulcus, medial cingulate cortex f. Insula, ventrolateral prefrontal cortex, middle occipital gyrus g. Superior occipital gyrus, medial temporal cortex, supplementary motor area, anterior cingulate cortex g. Inferior parietal lobule, superior parietal lobule
Santangelo et al. (2020)	a. Multivariate patterns exist in relation to ABM retrieval in HSAM participants & controls in: b. Significant effect of pattern distinctness comparing newer and older ABM’s in: c. Strength of pattern distinctness between newer & older memories for HSAM group increased in: d. ABM emotional valence-reliving quality not correlated with strength of pattern distinctness in:	a. L b. L c. L d. L	a. Dorsomedial prefrontal cortex, ventromedial prefrontal cortex, hippocampus b. Ventromedial prefrontal cortex (not the other two ROI) c. Ventromedial prefrontal cortex (not the other two ROI or for the control group) d. Dorsomedial prefrontal cortex, ventromedial prefrontal cortex, hippocampus
Mazzoni et al. (2019)	a. Areas activated during access vs. no memory: b. Areas activated during elaboration vs. no memory: c. Significant differences access versus elaboration d. Significant differences in elaboration versus access e. Brain areas common to access and elaboration (vs. no memory)	a. L b. L b. R c. L c. R c. B d. R d. B d. L e. L e. B	a. Cerebellum, middle frontal gyrus, precuneus, superior parietal lobule, lingual gyrus b. Middle temporal gyrus, inferior parietal lobule, superior temporal gyrus, inferior frontal gyrus, superior frontal gyrus, middle frontal gyrus, posterior cingulate gyrus b. Precuneus c. Lingual gyrus, precentral gyrus, medial frontal gyrus, fusiform gyrus c. Cuneus, posterior cingulate gyrus, inferior frontal gyrus, superior occipital gyrus, angular gyrus, superior parietal lobule c. Precuneus, middle frontal gyrus, cerebellum d. Superior temporal gyrus, inferior parietal lobule, transverse temporal gyrus d. Precuneus, middle frontal gyrus, superior frontal gyrus, precentral gyrus d. Posterior cingulate gyrus, angular gyrus, middle temporal gyrus, postcentral gyrus, inferior frontal gyrus, medial frontal gyrus, anterior cingulate gyrus e. Precuneus, middle occipital gyrus, middle frontal gyrus, superior occipital gyrus, superior frontal gyrus, superior temporal gyrus, middle cingulate gyrus, cerebellum, superior parietal lobule, cuneus e. Middle temporal gyrus
Santangelo et al. (2021)	a. Memory access > reliving (reliving had no increased activation over access) b. ABMs with higher positive emotional ratings showed greater activation in the: c. ABMs with decreased reliving ratings showed greater activation in:	a. B a. R a. L b. B b. L b. R c. R c. L	a. Supramarginal gyrus, superior temporal gyrus, angular gyrus, precuneus, superior occipital gyrus, insula, frontal orbital cortex, fusiform gyrus a. Middle occipital gyrus, a. Cuneus, posterior cingulate cortex, inferior temporal gyrus, middle frontal gyrus, middle temporal gyrus, thalamus b. Inferior frontal gyrus, frontal orbital cortex, insula, b. Precentral gyrus b. Thalamus c. Temporal pole, anterior cingulate cortex, frontal pole, ventromedial prefrontal cortex, supramarginal gyrus, superior parietal lobule, inferior frontal gyrus, middle frontal gyrus, temporoparietal junction, middle cingulate cortex, posterior cingulate cortex, c. Frontal orbital cortex, postcentral gyrus

*ROI* region of interest

#### ABM Access

Access is the moment a memory is reported to surface to consciousness. The temporoparietal junction, ventromedial prefrontal cortex, and dorsomedial prefrontal cortex on the left side were found to be selectively activated during HSAM access (Santangelo et al., 2018). Running a cvMANOVA, on the same data set, Santangelo et al. (2020) obtained a significant relationship between “pattern distinctness” (D measure) and older memories compared with newest memories for HSAM participants in the left ventromedial prefrontal cortex but not in left dorsomedial prefrontal cortex or the left hippocampus. During access, compared with recorded brain activity when no memory was being recalled, BB recruited left-sided (e.g., middle frontal gyrus, precuneus, and lingual gyrus) and posterior (e.g., cerebellum) brain areas (Mazzoni et al., 2019).

When comparing access vs. elaboration, significant activation was detected in the precuneus in both BB and GC (Mazzoni et al., 2019; Santangelo et al., 2021). On the left-side increases in activation were found in the thalamus and frontal (middle frontal gyrus), temporal (middle and inferior temporal gyrus), limbic (posterior cingulate cortex), and occipital lobes (cuneus) in GC (Santangelo et al., 2021). In BB the cuneus (right) and middle frontal gyrus (bilaterally) were also significantly activated (Mazzoni et al., 2019). BB additionally recruited many right-sided brain areas, including the superior parietal lobule.

#### Memory Elaboration

Elaboration-reliving is when a memory is remembered in its entirety. Reliving showed no detectable significant increases in activation, compared with access in GC (Santangelo et al., 2021). Compared with the no memory condition, elaboration resulted in increases in activation in the right precuneus and several left hemisphere structures (e.g., inferior frontal gyrus, inferior parietal lobule) in BB (Mazzoni et al., 2019). When elaboration was compared with access, BB displayed greater activation in structures across both hemispheres. Regions showing increases in activation were in the right hemisphere (e.g., superior temporal gyrus, inferior parietal lobule), bilaterally in the frontal lobe (i.e., middle and superior frontal gyri), and in the left hemisphere in the frontal (inferior frontal gyrus, medial frontal gyrus), parietal (postcentral gyrus, angular gyrus), temporal (middle temporal gyrus), and limbic lobes (posterior cingulate gyrus, anterior cingulate gyrus).

#### Overall Retrieval

HSAM individuals had greater neural activity than controls during overall retrieval in areas including, the bilateral angular gyrus and ventromedial prefrontal cortex, right dorsolateral prefrontal cortex and left temporoparietal junction (Santangelo et al., 2018). Mazzoni et al. (2019) found overall memory retrieval, compared with a no memory condition, recruited left side brain areas (e.g., the precuneus, cuneus, frontal gyrus, and temporal gyrus), and the bilateral middle temporal gyrus.

#### Behaviour Results

HSAM participants were excellent at retrieving memories using date cues (Mazzoni et al., 2019; Santangelo et al., 2021). Post-scanner verification of dates showed 100% accuracy in verifiable events (Santangelo et al., 2021). BB’s mean time to access (1816 ms) and elaborate (11,725 ms) memories was very fast (Mazzoni et al., 2019). HSAM groups were faster at accessing ABMs than controls for non-date cues and provided more detailed post-scanner descriptions (Santangelo et al., 2018). The same study found no between group-differences for self-report measures of emotional intensity or reliving rating for ABMs.

Overall, collated data from the limited number of functional neuroimaging studies reveal an involvement of a wide range of cortical areas and across all lobes during memory retrieval, in individuals that possess HSAM. Such widespread activity was observed in all stages of memory retrieval (i.e., access and elaboration-reliving), and behaviourally, speed of retrieval was very fast (< 2 s).

## Discussion

To our knowledge, this is the first systematic review on HSAM. The goal of this work was to collate the knowledge acquired from existing literature and to summarise what is currently known about the behavioural and neural basis of exceptional ABM. Fully understanding how exceptional memory functions could provide an alternative viewpoint to the study of human memory, that is more traditionally based on memory deficits (e.g., Cole et al., 2015; Rathbone et al., 2009).

The collated data presented in this systematic review leads to some interesting interpretations of how neurocognitive systems may sustain HSAM. Firstly, HSAM individuals and controls were found to be comparable for both number and quality of details described for autobiographical events dated closest to testing (LePort et al., 2016). This finding suggests that acquisition of information is not quantitatively or qualitatively enhanced, allowing us to claim that encoding processes in HSAM may be similar to the cognitive and metacognitive mechanisms of the normal population. It seems logical then that memory enhancement must occur in later memory stages (i.e., consolidation and retrieval) and these behavioural data encourages investigators to direct their attention to the forgetting processes in HSAM, that appear to be not in line with the expected pattern as in the classic Ebbinghaus’ forgetting curve (Ebbinghaus, 1885; Radvansky et al., 2022). For dates dating back one month or more from testing, HSAM individuals are vastly superior in their autobiographical recall (LePort et al., 2016), suggesting enhanced consolidation underlies the capacity to retain personal information. Following this line of reasoning, it seems HSAM individuals are not necessarily able to remember everything but instead, are *unable to forget* personal experiences. However, it must be noted that if additional measures during retrieval were recorded (e.g., response times or brain activations), between group differences may become evident at less than one week, contradicting the theory that HSAM involves normal encoding abilities. Furthermore, the aforementioned study (LePort et al., 2016), as with most that investigate ABM, assumed that all recollected ABMs described by participant’s were accurate, despite having no means to verify any claims. Perhaps even after one day, individuals with HSAM have more accurate memory representations. To address this uncertainty, wearable cameras during ABM encoding could be utilised in the future, allowing for accuracy of personal elements to be objectively measured, and the nature of HSAM processes to be better understood.

Moreover, the HSAM pass rate for the highly difficult PEQ also supports heightened consolidation, as it relies on dates of famous events (e.g., a date of a World Cup Final match) as memory cues (LePort et al., 2012; Santangelo et al., 2021). The perceived cultural importance of selected PEQ stimuli increases the likelihood that these events were initially encoded by people and thus is deemed a strong measure of whether individuals have retained or forgotten such information. Where those with normal memory have been shown to score close to zero on this task, individuals with HSAM must score a minimum of 50% (LePort et al., 2016) and some participants have been found to score over 90% accuracy (Talbot et al., 2022). Of course, the assumption that public events are known to all participants may not be fully warranted, and it might be useful to develop assessment tools, like the HMSQ (Mazzoni et al., 2019) that are more specifically tailored to individuals, to avoid failing to identify an exceptional case.

From an interpretational viewpoint, resting-state functional connectivity data highlighted in this review suggests that HSAM individuals may have better consolidation skills. Higher betweenness centrality and increased resting-state functional connectivity of the right orbitofrontal cortex and right lobule IX of the cerebellum were observed in BB (De Marco et al., 2021). The orbitofrontal cortex is believed to interact with the hippocampus during the formation of long-term memories (Ramus et al., 2007) and therefore could contribute to heightened consolidation in HSAM. In fact, greater resting-state connectivity of the hippocampus with other cortical and limbic structures (and cerebellar structures in some cases) was found to be the most consistent difference in HSAM (vs. controls) in this review (Ally et al., 2013; Brandt & Bakker, 2018; Daviddi et al., 2022a), and these differences could explain why forgetting is reduced in HSAM. According to the systems model of how memories are consolidated (see Squire et al., 2015), the hippocampus directs reorganisation of information to regions of the neocortex. This process is believed to transform a memory from labile to a more permanent memory trace that is eventually no longer dependent on the hippocampus; a stronger resting-state connectivity of the hippocampus with several other structures in HSAM could amplify this process. Crucially, whether this interpretation is true cannot be concluded in a study that lacks any kind of behavioural measures. Indeed, the risk that reverse inferences pose when interpreting neuroimaging data is well described (Poldrack, 2011). Future research should consider ways to test this explanation empirically.

Only one study (De Marco et al., 2021) has investigated graph theory-informed metrics of functional connectivity. This approach is complementary to the typical map’s representative of statistical modelling of regional signal; graph-theory indices can inform, amongst others, about computational centrality, integration, and segregation of regions via a pathway-based elaboration of correlational measures. Contrastingly to fMRI data, structural neuroimaging studies have shown no neuroanatomical differences in the size of the hippocampus related to HSAM (Ford et al., 2022; Gibson et al., 2022; Santangelo et al., 2021). However, structural differences were generally found to be highly inconsistent across the included studies. Furthermore, one study (Ally et al., 2013) used a blind participant, lowering the degree of confidence in the conclusions that can be drawn from neuroanatomical data.

Further potential insight on why information is better consolidated in HSAM comes from the only available resting-state group study (Daviddi et al., 2022a). Disrupted functional connectivity was observed in HSAM participants between the hippocampus and saliency network related brain regions (anterior cingulate cortex and bilateral insula). The authors refer to the salience network as “a core hub” that allows the detection of relevant stimuli present in the external environment, resulting in goal-directed behaviours as an outcome (Uddin, 2014). Furthermore, they observed decreased connectivity between the hippocampi and ventral frontoparietal regions (e.g., temporoparietal junction) that they describe as contributing to “deployment of attentional resources” (Corbetta et al., 2008). Enhanced functional connectivity of the hippocampus with sensory regions (e.g., inferior temporal cortex) was also observed. Taken collectively, the authors speculate that these findings could suggest that HSAM individuals are less able to discriminate or choose salient information, and this leads to greater encoding and consolidation of sensory information, regardless of how relevant it might be. As the authors clearly acknowledge themselves, this is an inference that cannot be confirmed using a resting-state study design, and thus they recommend further studies with behavioural measures. As memory consolidation is believed to occur partly during sleep (Cairney et al., 2014; Walker & Stickgold, 2004), we may expect that HSAM is linked with better sleep. However, Patihis (2015) found no significantly better patterns of sleep in HSAM individuals on self-reports measures of sleep quality (e.g., time taken to fall asleep). To ensure there are truly no meaningful differences in consolidation during sleep, additional research should consider using overnight physiological monitoring measures, such as a polysomnography, in the study of HSAM.

Based on the evidence that we have presented thus far, it appears a reasonable postulation that HSAM could be characterised by enhanced consolidation. However, the results of our systematic review reveal a lack of empirical evidence to explain *how* this enhanced consolidation may occur. The current HSAM literature cannot adequately explain the neurobiological processes that underlie this stage of memory. Looking to the wider literature, for several decades, research on both animals and humans has supported that emotional arousal contributes to consolidation (see McGaugh, 2013; McGaugh & Roozendaal, 2008). Hormones that are mediated by the amygdala and are released in response to stress (e.g., adrenaline and corticosterone) are thought to be involved in modulating whether or not a memory is retained (McGaugh, 2013). Perhaps then, enhanced remembering in HSAM is a result of a highly specialised activation of these modulatory systems. Similarly, HSAM individuals may share genetic or epigenetic markers that alter their memory ability. To our knowledge, no research exists that has explored these possibilities, though both could be promising avenues to explore in the future when working to understand better how HSAM occurs. Another possibility is that HSAM is linked to specialised aspects related to neurotransmission. Recent advancements in neuromolecular imaging, namely, the development of positron emission tomography (PET) radioligands that can pass the blood brain barrier, have allowed researchers to visualise the topography associated with specific aspects of neurotransmission. PET tracers have since been utilised in research and clinical studies spanning numerous disciplines (e.g., neurology and psychiatry) (Kilbourn, 2021). As an example, tracers have been created that are specific to D1 and D2 dopaminergic receptors (for a review see Kilbourn, 2021), and recently, it has been hypothesised that regional balance of D1 and D2 receptors is linked to cognitive functioning (for a review see Matzel & Sauce, 2023). Whether dopaminergic (or other types of) receptors contribute to cognitive functioning in HSAM is currently unknown, but using in vivo imaging techniques, like these, could provide direct insight into the biological mechanisms supporting superior memory. These suggestions, some of which we will be testing in our laboratory, are just a few possibilities to understand *if* and *how* consolidation is enhanced in HSAM.

Previously, some researchers (LePort et al., 2016) have hypothesised that underlying clinical conditions, such as obsessive–compulsive disorder (OCD), are prerequisites of HSAM that increase consolidation of memories through repetitive and habitual retrieval-practice (LePort et al., 2017). The OCD hypothesis derives from studies that have found that many HSAM participants have symptoms in line with OCD (e.g., LePort et al., 2012). Deliberate strategies, such as distributed practice or practice retrieval (see Schwartz et al., 2011), can strengthen memory, and thus, it is reasonable to infer that rehearsal and rumination over an autobiographical event could also help preserve this type of memory (LePort et al., 2016). According to this view, HSAM individuals are highly interested in their own personal memories, think about them frequently, and thus become excellent at remembering them.^2^ Consistent with this, research has shown that HSAM individuals enjoy thinking about their memories, reflecting on events whilst stuck in traffic (LePort et al., 2016) or whilst blow-drying their hair^3^ (Rodriguez McRobbie, 2017). Similarly, HSAM participants in our own laboratory have reported fears around forgetting information, and other individuals in the literature have stated that *understanding the importance of remembering* served as a turning point for which their own memory began to excel (Rodriguez McRobbie, 2017). The attachment to personal memories could explain why HSAM individuals were not enhanced at recalling memories less related to themselves in the Meta Test (LePort et al., 2017) or were found to be unable to remember what an interviewer was wearing after having sat for hours in front of them (Rodriguez McRobbie, 2017). Of course, the time required to rehearse every day of one’s life would be far too excessive for explicit rehearsal to be the sole process responsible for superior memory. The authors (LePort et al., 2016) acknowledge that HSAM individuals do not spend as much time practicing as other groups of individuals with enhanced memory, such as memory champions (Foer, 2011). Therefore, they suggest that passive rumination could underlie the strengthening of ABM. Our systematic review partially contradicts the OCD hypothesis. In fact, many of the participants identified did not have OCD symptomatology (Brandt & Bakker, 2018; De Marco et al., 2021; Mazzoni et al., 2019; Santangelo et al., 2021). In addition, in participants with high OCD scores, no correlation was found between higher Leyton Obsessional Inventory (LOI) scores (i.e., self-reported obsessional symptom scores) and faster memory access (Santangelo et al., 2018). Thus, our review highlights that more research is needed to clarify how a cognitive enhancement state and a clinical trait might interact in HSAM.

Palombo et al. (2018) have previously theorised that specialised memory consolidation in HSAM could be a result of an enhanced self-reference effect. The self-reference effect states that information involving the self will be better remembered (e.g., Betz & Skowronski, 1997; Klein, 2012), and this is thought to be due to easier integration of this information with pre-existing schematic representations of oneself (Burden et al., 2021; Conway, 2005; Conway & Pleydell-Pearce, 2000). Recent studies provide additional support to this theory: RS provided descriptions richer in semantic and episodic details for future events involving herself than matched controls, but she was comparable to controls for scenarios unrelated to herself (Gibson et al., 2022). In another recent study, RS excelled at semantic tasks, seemingly by attaching personal information to them (Ford et al., 2022). For example, her ability to recall the Harry Potter books word for word appears to be associated with a related ABM from the time she read it. In a similar vein, descriptions from HSAM individuals emphasise the fact that their memories are highly personal (Parker et al., 2006), and using the Meta Test, LePort et al. (2017) showed that people with HSAM only excelled at recalling memories related to themselves. This task also successfully demonstrated that HSAM individuals do not incidentally encode everything, providing additional support for the previously discussed theory that normal encoding processes are linked to exceptional ABM.

Our review found strong evidence that HSAM involves extraordinary retrieval. fMRI evidence shows HSAM involves, in part, an intense *overactivation* of common brain regions belonging to the ABM network (Maguire, 2002; Svoboda et al., 2006), including many temporoparietal and prefrontal areas (Mazzoni et al., 2019; Santangelo et al., 2018, 2021). Compared with controls, more than twice as many brain regions are activated during retrieval in HSAM (Santangelo et al., 2018), and this may explain why access and elaboration of autobiographical material is extremely quick (see Conway et al., 2003; Conway & Loveday, 2010 for normal populations). Daselaar et al. (2007) previously mapped ABM retrieval in healthy individuals, distinguishing access and elaboration. They found initial access is predominantly supported by anterior structures, including frontal and temporal brain areas (e.g., right prefrontal cortex) and later elaboration recruits posterior areas (e.g., visual areas and precuneus). In contrast, neural activation and functional connectivity data in this review appear to suggest an early recruitment of posterior areas during HSAM access (Mazzoni et al., 2019; Santangelo et al., 2018, 2021). These include the precuneus that is thought to play a role in visual imagery (Ahmed et al., 2018) and in retrieval of memories that are considered true (Addis et al., 2004). Interestingly, this is the exact opposite pattern observed in a SDAM (i.e., a syndrome involving an incapability of reliving personal events) sample (Palombo et al., 2018); SDAM individuals showed a reduction in activation in the right precuneus during an ABM retrieval task. Taken together, these findings suggest that it is the level of neural activity in deputed areas rather than recruitment of *novel* brain structures that may support exceptional retrieval. Similarly, unlike the reported shift of neural activation from left-to-right between the brain hemispheres during access and elaboration (Conway et al., 2003), BB showed widespread and bilateral activations during memory elaboration. Unlike the findings of other studies (Conway et al., 2001), BB’s left hemisphere remained activated during elaboration, both anteriorly and posteriorly (Mazzoni et al., 2019).

An additional point for discussion is that if HSAM is simply an enhancement of normal ABM, it would also be susceptible to false memories (Wade et al., 2007). Widely replicated data has demonstrated that distortions can influence both initial encoding (e.g., Findley, 2012) and post-encoding of memories (Mazzoni & Memon, 2003; for a discussion, see Mazzoni & Vannucci, 2007), leading to the presence of false memories. False memories are often said to be the product of the reconstructive nature of memory processes (Conway & Loveday, 2015) and are characterised by several “sins” (Schacter, 1999, 2021). Patihis et al. (2013) found a comparable frequency of false memories between HSAM individuals and controls, a finding that suggests that HSAM people may have an extraordinary *strength* of personal memories, supported by otherwise ordinary cognitive abilities. An important limitation of the Patihis et al. (2013) study should be noted before reaching any conclusion: the false memory assessments that were used (e.g., neutral single words in the DRM) were not autobiographical in nature. Our review evidences that it is clear that only ABM is exceptional in HSAM (Brandt & Bakker, 2018; Daviddi et al., 2022b; Frithsen et al., 2018; Gibson et al., 2022; LePort et al., 2012, 2017; Levine et al., 2019, 2021; Parker et al., 2006); it might still be possible, therefore, that when appropriate stimuli (i.e., personal events) are presented, fewer false memories are obtained compared with controls.

Our review did find some evidence of unique processing in HSAM. Mazzoni et al. (2019) suggested that ABM access can bypass the hippocampus. Many authors, however, have suggested that the hippocampus is essential to ABM (Maguire, 2002; Svoboda et al., 2006), particularly during access (Daselaar et al., 2007), and during retrieval of specific and general memories (e.g., Addis et al., 2004). Evidence from a task-based fMRI study investigating ABM retrieval revealed that for BB a broad range of left hemisphere brain areas are activated during access, including the ventrolateral prefrontal cortex (Mazzoni et al., 2019), that is thought to be related to the semantic contribution to an ABM (Jacques, 2012). The authors suggest that in HSAM an ABM becomes “*semanticised*” and, as demonstrated by Santangelo et al. (2018), semantic memory retrieval is significantly quicker than autobiographical recall; this finding could explain why recall of a semanticised ABM is significantly faster. Of course, this finding is based on a single case, and it must be emphasised that Santangelo et al. (2018) did find activity in the hippocampus during access, when non-date cues were used. This review revealed that no group studies have published fMRI data using a date task or have differentiated neural activations among additional features of retrieval, such as direct vs generative retrieval (Harris & Berntsen, 2019). Though an ABM network has been previously identified (Svoboda et al., 2006), meta-analyses of ABM studies have demonstrated that brain activations differ between studies and this variance indicates that ABM processes fluctuate across individuals. If the theory that HSAM is simply an enhancement of normal ABM is accurate, it would also be reasonable to assume then that not everyone achieves HSAM in the same way either. Furthermore, the notion of neural reserve (e.g., Stern, 2009) explains that there is a significant interindividual variability in the neural mechanisms that are engaged when different people perform the same task. These differences might increase the difficulty in correctly interpreting group data and when inferring the specific mechanisms that are responsible for HSAM. With this rationale, single-case studies may be the most effective way to explore HSAM and should be prioritised in the future.

At present, very little is known about the qualitative aspects of this retrieval, such as whether there is any neural specialisation linked to the amount of detail provided during retrieval, or the degree of semantic complexity that characterise the memories. It could be suggested that any differences in functional activations found between HSAM and controls during retrieval are a result of the increased amount of information retrieved in HSAM, rather than the brain functioning in a unique way. Activation levels do not always indicate expertise (Bernardi et al., 2013; Jeon & Friederici, 2016), however, and our review also found similar patterns of high neural activity during autobiographical retrieval in single-case reports that made within-subject comparisons. In the future, it could be informative to compare HSAM and controls on more recent memories, where the amount retrieved is closer matched. A fMRI study which measures what happens when the number of details between exceptional memory and normal memory are equated could be very beneficial to the field. We hypothesise that differences would still be observed, and the results could provide strong evidence that individuals with HSAM truly are superior. One may argue that qualitative differences are also the reason for resting-state distinctions between HSAM individuals and controls and thus should be considerations taken by researchers when interpreting results (see Heit, 2015 for a discussion on the topic of forward inference). Whilst this could be an explanation, our review did not find any empirical evidence to support that, from a hierarchical point of review, remembering takes priority (i.e., occurs more frequently or for longer) over any other process during resting state (e.g., planning and inner language). Collectively our review revealed that a lot is still unknown about the neural functioning of those with HSAM and that future research is needed to draw clearer conclusions about how the ability is supported.

Collated behavioural evidence supports that each HSAM participant in this review underwent extensive ABM assessment to support their categorisation as exceptional. The enhanced performance and test–retest reliability when providing personal memories leads us to define HSAM as *a rare ability involving very rapid, accurate, and extremely detailed retrieval of autobiographical memories, that is effortless, intrinsically tied to dates and that contrasts normal age-related decline.* Our review found HSAM manifests itself “spontaneously” that people with HSAM have a heightened trust in memory accuracy (Patihis, 2015), a more liberal response bias criterion (Frithsen et al., 2018), higher absorption, and fantasy proneness (Patihis, 2015), and possibly have a stronger associative memory for faces (LePort et al., 2017). Our synthesised results demonstrate that performance in tasks that measure other aspects of memory or cognition is entirely within normal age limits. Considering these findings, Roediger and McDermott (2013) present an interesting explanation of why HSAM individuals do not excel at other laboratory-based memory tasks. In line with meta-analytical findings (McDermott et al., 2009), the authors argue that the specificity of performance enhancement observed in HSAM reflects the retrograde versus anterograde distinction (i.e., ABM for life events and learning of new episodic information in the laboratory, respectively) that characterises episodic memory, with HSAM individuals showing superior levels of retrograde retrieval only. These two distinct forms of retrieval are tested with instruments that prompt different sets of sills, i.e., anterograde memory tasks require participants to engage in retrieval as well as encoding (as the material is new), while retrograde memory requires participants to engage in retrieval only (as it is assumed that encoding occurred in their autobiographical past). Clinical data also offer support to this explanation. It is well established that the systems underlying retrograde and anterograde memory are dissociable (Smith et al., 2013); patients with damage to certain brain areas cannot learn and retrieve new memories, but their ability to recall older autobiographical information remains intact. This separation in memory types could also explain why memory champions that possess a form of highly superior memory (Dresler et al., 2017; Foer, 2011; Maguire et al., 2002) only excel at laboratory-like tasks of remembering (Roediger and McDermot, 2013). Moreover, whilst both forms of memory retrieval (HSAM and memory champions) can be defined as superior, it should be emphasised that the latter have a “normal” memory that is extremely well-trained and that involves specific learning strategies (for a critical review on the use of strategies in the context of learning and cognitive plasticity, please see Lövdén et al. (2010)). Another possibility is that the self-referential component of ABM is what separates personal memory from the purely episodic memory system. Literature has shown that when the self is involved during encoding, people are better at remembering both past events (Stendardi et al., 2021) and imagined future events (Jeunehomme & D'Argembeau, 2021). How the role of the self could be related to HSAM has been considered in greater detail earlier in the discussion.

Overall, we argue that the only defining behavioural characteristics substantially supported in the literature are those we have described, including speed of retrieval, number of details remembered, and public event knowledge, that have been objectively measured. As this area remains largely under researched, mainly due to the low frequency of HSAM, future research could lead to further development of this description. This review has highlighted that in HSAM retrieval is vastly heightened, while memory consolidation is possibly enriched. Less is known about how encoding occurs, due to lack of neural data, but we hypothesise that it is comparable to general ABM and in this way is likely susceptible to false memories. The ultimate goal of understanding exceptional memory is to design therapeutic targets that could combat memory impairment. Functional neuroimaging (e.g., fMRI or functional near-infrared spectroscopy, fNIRS), neurophysiological (electroencephalography, EEG) and neuromodulation (transcranial magnetic stimulation, TMS) studies on HSAM could guide researchers to discover target areas, the stimulation of which could enhance ABM (Santangelo et al., 2022; for the first HSAM short report using TMS, see Talbot et al., 2022).

## Supplementary Information

Below is the link to the electronic supplementary material.

Supplementary file1 (DOCX 34 KB)

## Data Availability

Not applicable.

## Footnotes

1 Readers should note that when occupations are not described within the articles, this does not necessarily mean a HSAM participant is unemployed. News articles on the topic of hyperthymesia have reported that HSAM individuals work in an array of professions, including a professional violinist, radio news anchor and an actress (Stahl, 2014). Stahl, L. (2014). The gift of endless memory. CBS News. https://www.cbsnews.com/news/the-gift-of-endless-memory/

2 Listen to Correa & Nath (2022) for Podcast anecdotal evidence from a HSAM participant about exercising their own memory. Correa, D., & Nath, A. (Host). (2022-present). Marilu Henner and the Mysteries of Memory [Audio podcast episode]. Brain & Life podcast. Brain & Life. https://www.brainandlife.org/podcast/marilu-henner-and-the-mysteries-of-memory

3 Rodriguez McRobbie, L. (2017). Total recall: the people who never forget. The Guardian. Retrieved August 7, 2023, from https://www.theguardian.com/science/2017/feb/08/total-recall-the-people-who-never-forget.
