# Supplementary material for: Highly Superior Autobiographical Memory (HSAM): A Systematic Review
Source: Neuropsychol Rev. 2024 Feb 23;35(1):54–76. doi: 10.1007/s11065-024-09632-8 (PMC11965258; doi:10.1007/s11065-024-09632-8)
Supplement: Supplementary file 1 — Supplementary file1 (DOCX 34 KB) [file 11065_2024_9632_MOESM1_ESM.docx]

**Highly Superior Autobiographical Memory (HSAM): A Systematic Review**

Jessica Talbot^1^*, Gianmarco Convertino^1^, Matteo De Marco^2^, Annalena Venneri^2,3^, Giuliana Mazzoni^1,4^

*^1^ Department of Dynamic and Clinical Psychology, and Health Studies, University La Sapienza, Rome, Italy*

*^2^ Department of Life Sciences, Brunel University London, Uxbridge, United Kingdom*

*^3^Department of Medicine and Surgery, University of Parma, Parma, Italy*

*^4^ Department of Psychology, University of Hull, Hull, United Kingdom*

* Corresponding author:

Jessica Talbot, Department of Dynamic and Clinical Psychology, and Health Studies, Via degli Apuli, 1, 00185, Roma RM University La Sapienza; *e-mail*: jessica.talbot@uniroma1.it

Example search String:

PubMed (2006 – present day)

("superior"[Title/Abstract] OR "exceptional"[Title/Abstract] OR "extraordinary"[Title/Abstract] OR "hyperthym*"[Title/Abstract] OR "savant"[Title/Abstract]) AND ("autobiograp*"[Title/Abstract] OR "personal"[Title/Abstract] OR "hyperthym*"[Title/Abstract]) AND ("memor*"[Title/Abstract] OR "retriev*"[Title/Abstract] OR "recall*"[Title/Abstract] OR "recogn*"[Title/Abstract] OR "encod*"[Title/Abstract] OR "rememb*"[Title/Abstract] OR "mnem*"[Title/Abstract] OR "mnes*"[Title/Abstract] OR “recollect*"[Title/Abstract])

Supplementary Table 1. Main behaviour results from HSAM studies.

| **Ref.** | **Neurocognitive measure** | **Task names** | **Main findings** | **Rank** |
| --- | --- | --- | --- | --- |
| (Parker et al., 2006) | a) ABM  b) overall memory  c) visuospatial memory  d) recognition memory  e) attention/working memory  f) olfactory function  g) face perception  h) executive function & reasoning  i) anterior left hemisphere tests  j) organisationally demanding  k) verbal memory  l) semantic memory  m) manual dexterity  n) language  o) calculations  p) intelligence | a) Dates Task, Easter Dates Task, Famous Events Quiz, Remembering previously discussed dates, AMT (ABM and semantic memory components)  b) General Memory Index on WMS-R  c) Visual Memory Index on WMS-R, Visual Paired Associates (immediate & delayed)  c) Picture Completion, Picture Arrangement, Block Design, Object Assembly, ROCFT Copy, Hooper Visual Organization  d) Word Recognition (Warrington Test) & Word Recognition from CVLT  d) Face Recognition, Warrington Test  e) Digit Span (WAIS-R), Attention/ Concentration Index (WMS-R)  f) Smell Identification Test, Sensory-Perceptual exam  g) Benton Face Test  h) Perseverative Responses (Concept formation & Shifting from WCST), Executive Functions from HCT, Analogical Reasoning (WAIS-R)  h) Written Fluency, Oral Fluency: Letters, Oral Fluency: Category, Ruff Figural Fluency, Trails A, Trails B, Stoelting Stroop Test  i) Motor Speed, Right Dominant Hand, Dysnomia from Boston Naming  j) Recall of Wordlist from CVLT, Recall of Complex Figure (both delays)  k) WMS-R Verbal Memory (immediate and delayed)  l) WAIS-R Information  m) Grooved Peg, Right, Left  n) Reading Level, Spelling Level, Vocabulary, Comprehension  o) Arithmetic Level, Arithmetic  p) WAIS-R (full-scale IQ, verbal IQ, performance IQ) | a) overall performance excellent / perfect  b) overall performance excellent  c) overall performance excellent  c) overall performance normal  d) overall performance excellent  d) performance was impaired  e) overall performance excellent  f) overall performance excellent  g) performance was enhanced  h) performance was impaired  h) overall performance normal  i) performance was impaired  j) Performance was impaired  k) overall performance normal  l) overall performance normal  m) overall performance normal  n) overall performance normal  o) overall performance normal  p) all scores above average / normal range | a) enhanced  b) enhanced  c) enhanced  c) normal  d) enhanced  d) inferior  e) enhanced  f) enhanced  g) enhanced  h) inferior  h) normal  i) inferior  j) inferior  k) normal  l) normal  m) normal  n) normal  o) normal  p) normal |
| (LePort et al., 2012) | a) ABM  b) associative memory  c) visual memory  d) attention/working memory  e) episodic memory  f) verbal memory | a) AMT  b) Names to Faces  c) Visual memory task  c) Visual reproduction  d) Digit span forwards and backwards  e) WMS-III Logical Memory Subtest – recognition  e) WMS-III Logical Memory Subtest – free recall  f) Verbal paired associates | a) overall performance superior to controls  b) overall performance superior to controls  c) overall performance superior to controls  c) overall performance comparable to controls  d) overall performance comparable to controls  e) overall performance comparable to controls  e) overall performance superior to controls  f) overall performance comparable to controls | a) enhanced  b) enhanced  c) enhanced  c) normal  d) normal  e) normal  e) enhanced  f) normal |
| Patihis et al., 2013) | a) false memories | a) DRM  b) Misinformation Task  c) non-existent news-footage paradigm | a) false memory rate comparable between groups  (HSAM vs. controls, high vs. low PEQ scores)  a) HSAM participants recognised more previously presented words correctly  b) HSAM individuals had significantly more overall false memories than controls  b) source-confirmed false memory rate comparable between groups (HSAM vs. controls, high vs. low PEQ scores)  c) tendency to report false footage seen before, number of false details reported, and susceptibility to semiautobiographical false memories comparable between groups (HSAM vs. controls, high vs. low PEQ scores) | a) normal  a) enhanced  b) inferior  b) normal  c) normal |
| (Santangelo et al., 2021) | a) ABM | a) Random Dates Quiz and Public Events Quiz | a) GC’s performance was found to stable, and in HSAM range, at 75 and 80 years old, & his performance was comparable to younger HSAMs, & the majority of ABM details episodic | a) enhanced |
| (LePort et al., 2016a) | a) ABM | a) Dates Task | a) HSAM individuals provided more ABM details (quantity) at 1 month, 1 year and 10 years, quality of internal details at 1 month onwards and for the past 7 days higher for HSAM  a) number of internal details at 1 week, quantity of details over a 7-day period, and quality of internal details at 1 week comparable between groups | a) enhanced  b) normal |
| (Patihis, 2015) | a) openness to absorbing new experiences, synaesthesia  b) fantasy proneness  c) empathy  d) high arousal emotions  e) sleep  f) intelligence  g) critical thinking  h) flexible thinking  i) beliefs about memory | a) TAS  b) CEQ  c) BES  d) High Arousal Emotions - Likert scale  e) Sleep Diaries  f) SAT scores  g) 9 item critical thinking questionnaire  h) The Flexible Thinking Scale  i) Memory for belief questions | a) HSAMs scored higher on: overall TAS than controls, on the sum of the 3 questions relating to synaesthesia, and on Q 17 “different colours have distinctive and special meanings for me”  a) higher scores on overall TAS, and Q17 positively correlated with 10 Dates Quiz scores  b) HSAMs scored higher on CEQ than controls, and higher scores on overall CEQ positively correlated with 10 Dates Quiz scores  c) BES scores comparable between groups  d) frequency of high arousal emotions felt were comparable between groups  e) sleep quantity / quality, frequency of daytime naps, number minutes napped comparable  f) overall SATS scores, or on maths / reading only scores were comparable between groups  g) critical thinking scores were comparable  h) HSAMs had lower flexible thinking scores, and “tolerance for ambiguity” scores  i) HSAM groups and controls have similar beliefs about human memory fundamentally works  i) HSAM had higher trust that their memory was reliable compared to memory researchers | a) enhanced  b) enhanced  c) normal  d) normal  e) normal  f) normal  g) normal  h) inferior  i) normal  i) higher |
| (LePort et al., 2016b) | a) associative memory  b) mental imagery  c) visual short-term memory  d) visuospatial ability  e) semantic memory, narrative skills, verbal fluency  f) emotional intensity / ABM  g) inhibition  h) pattern separation  i) verbal learning and memory  j) ABM | a) Face-Name-Occupations Task  b) Mental Imagery Task  c) Visual Patterns Task  d) Progressive Silhouettes Task  e) Script Generation Task  f) Three Phase Story  g) Stroop Task  h) Mnemonic Similarity Task  i) CVLT  j) Meta Test | a) overall HSAMs superior to controls  b) overall HSAMs comparable to controls  c) overall HSAMs comparable to controls  d) overall HSAMs comparable to controls  e) overall HSAMs superior to controls  f) HSAMs recalled fewer peripheral details than controls in Phase 2  g) overall HSAMs comparable to controls  h) overall HSAMs comparable to controls  i) HSAMs scored more for total recall of List A  i) groups comparable overall at word list recall  j) HSAMs more accurate at recalling exact date previous testing sessions happened, and better at recalling personal ABM’s  j) ability to recall order of cognitive tests, and recall of ABMs shared by the experimenter comparable between groups | a) enhanced  b) normal  c) normal  d) normal  e) enhanced  f) inferior  g) normal  h) normal  i) enhanced  i) normal  j) enhanced  j) normal |
| (Daviddi et al., 2022b) | a) divergent thinking  b) divergent thinking  c) convergent thinking  d) attention / working memory | a) AUT  b) CT  c) RAT  d) Digit Span | a) overall HSAMs comparable to controls  b) overall HSAMs comparable to controls  c) overall HSAMs comparable to controls  d) overall HSAMs comparable to controls | a) normal  b) normal  c) normal  d) normal |
| (Ford et al., 2022) | a) semantic memory / ABM  b) semantic memory / ABM | a) Calendar Dates Task  b) Harry Potter Task | a) RS had significantly higher overall accuracy, slower median reaction time (indicates, unlike controls, she was not guessing), and scored above chance for temporal distance, year position, 2005, 2010, and 2015 items  b) RS more accurate & faster at detecting which sentence appeared earlier, she scored above chance for start and end book sentences, and close and distant sentence pairs | a) enhanced  b) enhanced |
| (Frithsen et al., 2018) | a) response bias and recollection rates | a) Remember / Know paradigm, Source Test | a) no meaningful differences between HSAMs / controls for overall discrimination of old or new words / “discrimination based on recollection” / familiarity, or response bias based on recollection / familiarity  b) overall HSAM participants had a lower response bias criterion | a) normal  b) lower |
| (Levine et al., 2019) | a) predicting / remembering emotions about events | a) Election Questionnaire | a) overall HSAM individuals comparable to controls at predicting & recollecting emotional responses to a government election | a) normal |
| (Levine et al., 2021)  Study 1. | a) remembering facts/ feelings about events | a) Election Questionnaire | a) HSAM individuals more accurate at remembering elections facts than controls (only small improvement to controls)  a) accuracy at remembering emotions and intensity of emotions post-election comparable | e) enhanced  a) normal |
| (Gibson et al., 2022) | a) intelligence  b) language  c) visuospatial  d) motor function  e) attention / working memory  f) executive functioning  g) visual memory  h) recognition memory  i) prospective memory  j) empathy  k) ABM  l) ABM future thinking  m) future thinking | a) NART Estimated IQ, WASI-II, Raven’s Advanced Progressive Matrices  b) Graded Naming Test, WASI-II VCI, semantic fluency (animals)  c) VOSP Incomplete Letters, WASI-II PRI, Rey Complex Figure (copy)  d) Grooved Pegboard (right & left)  e) Test of Everyday Attention, Digit Span (forwards & backwards)  f) Phonemic Fluency, Colour-Word Test, Hayling Sentence Completion,  Trail Making Test (A&B), Brixton Spatial Anticipation Test, D-Kefs Tower Test, Ideational Fluency  g) Spatial Location & Design Content (immediate and delayed)  h) abstract designs, topographical scenes & faces  i) The Brief Assessment of Prospective Memory  j) self-report measure  k) AI  l) A-AI  m) Narrative Scene Construction – Cinderella and Cookie Theft | a) RS had overall average intelligence  b) performance was within average range  c) performance was average  d) right score was average & left low average  e) overall performance was normal  f) overall performance within average range  g) overall performance was average  h) overall performance was average  i) RS’s ability to complete activities of daily living was normal of her age category  j) average at “experiencing others’ emotions”  j) ability to “comprehend emotions of others” reported as poor  k) number of internal / external details at Mid Childhood, Adolescence, Early Adulthood (Event 2) & Previous year, self-report of ratings of “Visualisation, Emotional Change, Current importance or Past Importance” comparable between groups  k) controls shared more external details in Early Adulthood Event 1 than RS  l) overall RS provided more internal & external details for past and future events  l) no between group differences in self-reported ratings of “Level of Detail, Emotionality, Personal Significance or Temporal Distance”  m) results were comparable for Total Words, Novel Ideas and Generation Quality between groups  g) RS repeated herself more | a) normal  b) normal  c) normal  d) normal  e) normal  f) normal  g) normal  h) normal  i) normal  j) normal  j) inferior  k) normal  k) inferior  l) superior  l) normal  m) normal  m) inferior |
| (Mazzoni et al., 2019) | a) ABM  b) intelligence  c) figural memory  d) recognition memory  e) visual memory  f) attention / working memory  g) face perception  h) executive function  i) language | a) Dates Tasks, Birthday Memory Test  b) WAIS-III  c) Figural Memory Task  d) CVLTII  e) Visual Reproduction (immediate and delayed),  e) Rey Figure Copy and Rey Memory task    f) Digit Span (forwards and backwards)  g) Benton Task  h) Mental Control, Trail Making, Stroop tasks  i) WAIS-R comprehension test, Vocabulary test, D-KEFS Verbal Fluency  i) Boston Naming Test | a) BB demonstrated an excellent ability to recall ABMs in response to dates as cues, retrieval was much faster for dates (BB 1.8 seconds), and he could recall more birthdays than controls, accuracy for details that could be verified was 98%  b) BB’s IQ was in the top 90^th^ percentile  c) performance was perfect  d) long delay Yes-No and forced recognition were perfect  d) performance average in other areas of task  e) performance was excellent  e) performance was above average  f) performance was excellent  g) performance was above average  h) Performance was above average / excellent  i) Performance was above average / excellent  i) Performance was average | a) enhanced  b) enhanced  c) enhanced  d) enhanced  d) normal  e) enhanced  e) normal / enhanced  f) enhanced  g) normal/ enhanced  h) enhanced  i) enhanced  i) normal |
| (Ally et al., 2013) | a) intelligence  b) overall memory  c) ABM | a) WAIS (4^th^ Edition)  b) WMS-4  c) Dates Interview, semi-structured ABM questionnaire, | a) overall performance was average  b) overall performance was average  c) from age 11 onwards accuracy in recalling ABMs from dates from each year of his life near perfect, from adolescence most details reported are episodic in nature, consistency when dates repeated was 100% | a) normal  b) normal  c) enhanced |
| (Brandt & Bakker, 2018) | a) intelligence  b) overall memory  c) prospective memory  d) empathy and friendship  d) ABM | a) WAIS (4^th^ Edition)  b) WMS, ROCFT  b) President’s Test, Recognition of Celebrity Faces, Oscar Test, Date Significance, Fact Retrieval  c) remembering to return an item borrowed  d) Empathy Questionnaire, Friendship Questionnaire  d) Autobiographical Memory Interview, Personal Residence’s Inventory | a) overall performance was average  b) overall performance was average  b) overall performance was excellent  c) overall performance was average  d) overall results were average  d) overall performance was excellent | a) normal  b) normal  b) enhanced  c) normal  d) normal  d) enhanced |
| *Abbreviations:* Autobiographical Memory Test (AMT), Wechsler Memory Scale – Revised (WMS-R), Wechsler Memory Scale – Third Edition (WMS-III), The Rey–Osterrieth Complex Figure Test (ROCFT), California Verbal Learning Test (CVLT), Wechsler Adult Intelligence Scale Revised (WAIS-R), Wechsler Adult Intelligence Scale Fourth Edition (WAIS-4^th^), Deese-Roediger-McDermott task (DRM), Tellegen Absorption Scale (TAS), Creative Experience Questionnaire (CEQ), Basic Empathy Scale (BES), Scholastic Assessment Test (SAT), National Adult Reading Test (NART), Wechsler Abbreviated Scale of Intelligence – Second Edition (WASI-II), Verbal Intelligence Index (VCI), Perceptual Reasoning Index (PRI), Delis-Kaplan Executive Functioning Systems (D-KEFS) Proverb Test, Autobiographical Interview (AI), Adapted Autobiographical Interview (A-AI), Alternative Uses Task (AUT), Remote Association Task (RAT), Consequences Task (CT), Intellectual Quotient (IQ), Visual Object and Space Perception (VOSP). | | | | |
